# Significance of heat transfer rate in water-based nanoparticles with magnetic and shape factors effects: Tiwari and Das model

**DOI:** 10.1038/s41598-023-42480-9

**Published:** 2023-09-19

**Authors:** Syed Asif Ali Shah, Shumaila Kanwal, Muhammad Idrees, Asif Mahmood, Irfan Mahmood, Ali Akgul, Abdul Bariq

**Affiliations:** 1 Department of Mathematics and Statistics, The University of Lahore, Lahore, Pakistan; 2 Chemical Engineering Department, College of Engineering, King Saud University, Riyadh, Saudi Arabia; 3 Department of Mathematics, College of Science, Shanghai University, Shanghai, China; 4 Department of Computer Science and Mathematics, Lebanese American University, Beirut, Lebanon; 5 Department of Mathematics, Leghman University, Mehtarlam City, Laghman 2701 Afghanistan

**Keywords:** Engineering, Mathematics and computing, Nanoscience and technology

## Abstract

Nanofluids are implementable in a variety of applications, such as heat exchangers, the healthcare sector, the cooling of various devices, hybrid-powered machines, microelectronics, power plants, chemical processes, astronomical technology, cancer treatment, etc. Nanofluids also have enhanced heat transmission and thermal efficiency. The heat radiation of nanoparticles and the natural-convective flow of electrically conducting nanofluids over the rotating disk using Darcy Forchheimer’s porous media, thermal radiation is investigated in this paper. The nanoparticles titanium dioxide and single-walled carbon nanotubes are taken into account with base fluid water. The main goal of this investigation is to enhance heat transfer in nanofluids. The mathematical solution for the model has been obtained through the utilization of cylindrical coordinates. The flow model, which forms the basis of the investigation, is constructed around partial differential equations (PDEs). To address the inherent nonlinearity of these PDEs, physical similarities are employed to transform them into ordinary differential equations (ODEs). Subsequently, the fourth-order Runge–Kutta technique is employed via Matlab to solve these ODEs. The graphical examination of the velocities and temperature with various parameters is an exquisite display of scientific artistry. The magnetic field component is anticipated to exhibit an inverse correlation with velocities, while the temperature profile is expected to surge with the rise of the nonlinear mixed convection parameter. Additionally, the skin friction and Nusselt number are meticulously computed and presented in a tabular format, adding a touch of elegance to the already breathtaking analysis. By boosting the radiation parameter, the Nusselt value declined. Moreover, it is observed that the nanofluids having a laminar nanoparticle shape have a greater heat transfer rate.

## Introduction

In recent years, nanofluids have garnered a considerable amount of interest owing to their utilization in engineering and industrial sectors. Choi and Eastman^1^ made the initial nanofluid. To improve the thermophysical characteristics, other authors add their work in different ways of research. Overall, it is obvious that the employment of nanoparticles is essential for the industrial conditioning needed in the last stage of material production, tumor treatment, etc. The research of rotating disk-induced flows is widely used in many different applications, such as engines for gas turbines and motors, biomedical fields, spinning air filters, petroleum sciences, and food recent advancements. The study of these flows has experienced a recent surge in interest. Von-Karman^2^ conducted an important study on the continuous flow produced by the consistently high spinning of an infinite disk. Cochran^3^ studied the flow because of the rotating disk and he was able to asymptotically solve the stable hydrodynamic issue by utilizing the Von-Karman modifications. Ramzan et al.^4^ delved into the intricate impact of mixed convection in the presence of a magnetic field. Ganvir et al.^5^ expounded on the fascinating properties and thermal transmission rate in nanofluids. They illuminated the multifaceted relationship between temperature and matter, elucidated the ingenious use of surfactants in stabilizing nanoparticles, explicated the role of convection in heat transfer, elaborated on the diverse effects of thermal characteristics, and expounded on the intriguing influence of particle size. The calculation of heat transfer on the surface is intricately linked to the thermal conductivity and the thickness of connected layers.. Sadiq et al.^6^ analyzed thermal transfer for an axially symmetric nanofluid flow by Das and Tiwari model that was imposed parallel to a spiraled disk that is uniformly spinning and spreading radially.

Mustafa et al.^7^ studied the nanofluid flow of ferromagnetic Fe$_3$O$_2$ particles with base fluid suspended in a homogenous suspension over the rotating disk. Their research shows that by boosting the actual volume fraction of magnetic nanoparticles, the rate of surface temperature distribution can be enhanced. Reza-E-Rabbi et al.^8^ investigated the movement of multiphase fluid flow over a stretching sheet in the presence of nanoparticles using numerical models. They observed that the heat transfer c profiles are increased as a result of an elevation in the porous parameter. The effect of the thickness of connecting layers on thermophysical characteristics was covered by Lenin et al.^9^. They evaluated that their research is helpful for the efficiency of thermal properties and use in their profitable applications. In the midst of the presence of both fundamental materials, Ali et al.^10^ explored the profound significance of fluctuating nanomaterial radii for the non-Newtonian stream of nanofluid instigated by an expanding sheet. The study of magneto-hydrodynamics (MHD) steady flow in a Porous medium filled with Copper nanoparticles with base fluids water was conducted by Hayat et al.^11^. They meticulously examined the impact of partial slip and heat radiation. Shah et al.^12^ studied MHD nanofluid flow across the Riga plate. In this paper, they analyzed that the heat transfer rate enhanced for magnetic and thermal radiation parameters. Jamshed et al.^13^. conducted their research by incorporating two distinct types of nanofluids; one composed of titanium oxide engine oil and the other composed of copper engine oil. Their results demonstrate that a rise in both the Reynolds and Brinkman numbers leads to a notable augmentation in the system’s entropy. Nayak et al.^14^. examined the effect of Darcy Forchheimer on velocity and temperature using the Tiwari–Das model. They analyzed that thermal profile boosted for larger inputs of thermal radiation parameter.

To regulate the motion of nanofluid, a numerical method for mass and heat transfer enhancement/reduction is defined, which is studied with reactive chemicals flow passing a stretched surface that is inclined, by Reza-E-Rabbi et al.^15^. Ramzan et al.^16^ analyzed the effects of velocity and thermal slip variables on the magnitude of the velocity and Brownian motion coefficient by the Tiwari–Das model. They concluded that the temperature and concentration profiles increased for the thermophoretic parameter. Haq et al.^17^ studied the two-dimensional radiative mixed convective flows of nanofluid and non-Darcy porous substances that appeared on a wavy inclined surface. It is analyzed that an increase in thermophoresis and Brownian motion parameters yields a decrease in the rate of heat transfer. Ali et al.^18^ evaluated the motion of chemically linked species using the Arrhenius activation energy. In profitable and technological sectors of the industry, such as lubrication systems, cancer therapy, and solar cell enhancement, nanofluids are being used more and more. Sreedevi et al.^19^ conducted a numerical analysis using the Tiwari–Das model to investigate the temperature exchange and fluid flow characteristics of water-Ag-based reactive nanofluids on the inner part of a square hollow. The study was carried out under typical conditions on the horizontal walls and temperature-equivalent conditions on both sides of the walls.

When free/natural convection and enforced convection techniques work with each other to transport heat, this is known as mixed convection in the field. Safdar et al.^20^ studied mixed-convection MHD nanofluid flow across a stretched surface. They observed that the temperature profiles enhanced for higher input of radiation parameter. Xu^21^ investigated the spinning disk-induced mixed convection of a hybrid nanofluid. They simulate the situation, and a basic homogeneous flow model that explains nanofluids with nanoparticles of several types is constructed. Reza-E-Rabbi et al.^22^ analyzed the properties of fluid flow and heat transmission. They determined that a linear pattern is used to estimate the radiative heat flux. Bachok et al.^23^ studied the nanofluids flow with heat transfer across the spinning disk by Das and Tiwari model and the research outcomes of this research can be applied to the development of an efficient air conditioning network for electrical parts, thereby assisting in the creation of specific operating settings that is simultaneously efficient and secure. Fallah et al.^24^ analyzed that their research could be applied to rotating MHD energy sources for future space systems and heat conversion techniques for nuclear-powered spacecraft. Gamachu and Ibrahim^25^ analyzed that a higher volume percentage of alumina and silver nanoparticles can manage the distribution of temperature and concentration in mixed convection flow of hybrid nanofluid over the rotating disk.

Reza-E-Rabbi et al.^26^ analyzed the impact of heat radiation on MHD Casson nanofluid flow across a stretched sheet. They examined that the streamlines declined with the increase of the magnetic parameter. Prasad et al.^27^ observed a spinning disk with no mass flux and mixed convection Williamson flow of nanofluids. They analyzed the high amount of the heat buoyancy term increases the velocity and the concentration buoyancy factor decreases the velocity. Ahamd et al.^28^ observed the Maxwell nanofluid mixed convective flow induced by the rotating cylinder and also they noted that the swirl velocity and temperature were decreased and axial velocity was enhanced by greater values of the buoyancy and mixed convection variable. Dinarvand and Pop^29^ conducted a study on the laminar free convective flow and thermal transmission in an electrically conducting nanofluid of copper/water across a spinning downward-pointing vertical cone by utilizing the Tiwari–Das nanofluid technique. Their findings were quite intriguing. Rawat et al.^30^, on the other hand, delved into the study of MHD Cu-Water nanofluid flow, using Buongiorno’s model in the existence of heat radiation and chemical processes. They concluded that as the thermal relaxation parameter increases, both temperature and concentration curves decline. Finally, Mustafa et al.^31^ explored the impacts of MHD on the mixed convection flow of nanofluid particles in a stagnation area across a vertical plate, using copper and alumina nanofluid. Their findings were quite remarkable and insightful.

It is observed from the above literature that no one looked into the nanofluid flow over the rotating disk with the Tiwari and Das model. The novelties of this research include: (1) The impact of linear/nonlinear mixed convection is analyzed. (2) The impact of heat radiation is computed. (3) Tiwari–Das model is considered. (4) The nanoparticles TiO$_{2}$ and *SWCNT* are taken into and base fluid is water. (5) The slip parameters are examined. (6) The effect of different nanoparticles shape factors is analyzed. Moreover, The current study will address the following issues:

- What will be the impacts of magnetic, Darcy-Forchheimer, and mixed convection parameters on primary and secondary velocities?
- What will be the trend of temperature profile against shape factors, magnetic, thermal radiation, and Darcy-Forchheimer parameters?
- What will be the influence of magnetic, thermal radiation, Darcy-Forchheimer, shape factors, and mixed convection parameters over skin friction number and Nusselt number?

## Mathematical formulation

**Figure 1:**
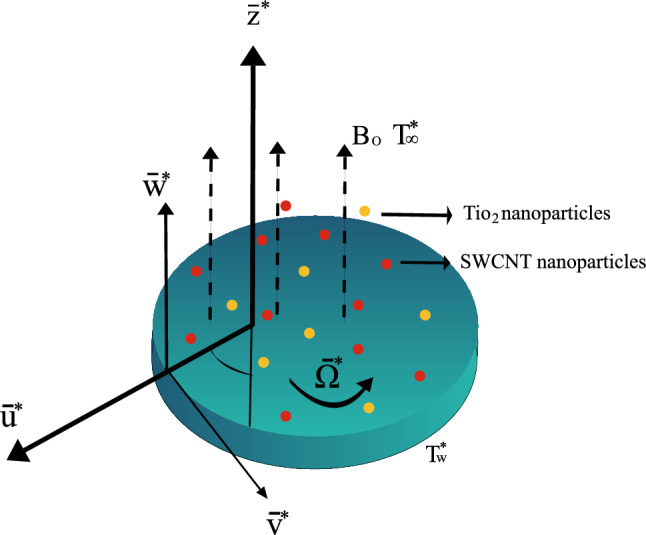
Geometry of the nanoparticles flow.

If $(\bar{r^*}, \varphi , \bar{z^*})$ is the collection of spherical coordinates, and the disk is positioned at $\bar{z^*}=0$ and the flow is generated by the disk rotating in the direction of the positive $\varphi$ at a constant angular velocity of $\bar{\Omega ^*}$. The fluid flow of nanoparticles expands over an infinitely large rough disk. The nanofluid flow velocity’s components are $\bar{u^*}$, $\bar{v^*}$ and $\bar{w^*}$ in the orientations of $(\bar{r^*}, \varphi , \bar{z^*})$. The ambient temperature, indicated by $(T^*)$, is reached a significant space from the boundary while the wall temperature is maintained consistently at $T^*_{\infty }$ = $(T^*_w)$. The partial slip situations are considered appropriate since the basic level of roughness at the disk is assumed to be lower than the difference among boundary layers. A constant $(B_o)$ magnetic field that acts axially. The magnetic field is likely to be omitted because the magnetic Reynolds ratio is so negligible. The flow problem is depicted in Fig. 1.

The following governing equations are given in vector form^32,33^

1 $$\begin{aligned} \nabla . V= & {} 0, \end{aligned}$$

2 $$\begin{aligned} \left( V. \nabla \right) \bar{u^*}= & {} -\frac{1}{\rho _{nf}} \nabla p + \frac{\mu _{nf}}{\rho _{nf}} \nabla ^2 V -\frac{\sigma _{nf}}{\rho _{nf}} B^{*2}_{0} \bar{u^*} +\frac{1}{\rho _{nf}} \bar{g^*}\left[ \Lambda _{1} (\bar{T^*}-T^*_{\infty })+(\Lambda _{2} (\bar{T^*}-T^*_{\infty })^{2}\right] -F\bar{u}^{*2}, \end{aligned}$$

3 $$\begin{aligned} \left( V. \nabla \right) \bar{v^*}= & {} \frac{\mu _{nf}}{\rho _{nf}} \nabla ^2 V -\frac{\sigma _{nf}}{\rho _{nf}} B^{*2}_{0} \bar{v^*} +\frac{1}{\rho _{nf}}\bar{g^*}\left[ \Lambda _{1} (\bar{T^*}-T^*_{\infty })+(\Lambda _{2} (\bar{T^*}-T^*_{\infty })^{2}\right] -F\bar{v}^{*2}, \end{aligned}$$

4 $$\begin{aligned} \rho _{nf} \left( V. \nabla \right) w= & {} - \nabla p + \mu _{nf} \nabla ^2 w, \end{aligned}$$

5 $$\begin{aligned} \left( V. \nabla \right) T= & {} \alpha _{nf} \nabla ^{2} T - \nabla . q_r. \end{aligned}$$

The equation system can be converted into consecutive approaches using the model of Tiwari–Das^31^.

6 $$\begin{aligned}{} & {} \frac{\partial \bar{u^*}}{\partial \bar{r^*}}+\frac{\bar{u^*}}{\bar{r^*}}+\frac{\partial \bar{w^*}}{\partial \bar{z^*}}=0, \end{aligned}$$

7 $$\begin{aligned}{} & {} \left( \frac{\partial \bar{u^*}}{\partial \bar{r^*}} \bar{u^*}-\frac{\bar{v}^{*2}}{\bar{r^*}}+ \frac{\partial \bar{u^*}}{\partial \bar{z^*}} \bar{w^*} \right) =-\frac{1}{\rho _{nf}}\frac{\partial p^*}{\partial \bar{r^*}}+\frac{\mu _{nf}}{\rho _{nf}}\left( \frac{\partial ^2 \bar{u^*}}{\partial \bar{r}^{*2}}+\frac{1}{\bar{r^*}}\frac{\partial \bar{u^*}}{\partial \bar{r^*}}- \frac{\bar{u^*}}{\bar{r}^{*2}}+\frac{\partial ^2 \bar{u^*}}{\partial \bar{z}^{*2}} \right) \nonumber \\{} & {} \quad -\frac{\sigma _{nf}}{\rho _{nf}} B^{*2}_{0} \bar{u^*} +\frac{1}{\rho _{nf}}\bar{g^*}\left[ \Lambda _{1} (\bar{T^*}-T^*_{\infty })+(\Lambda _{2} (\bar{T^*}-T^*_{\infty })^{2}\right] -F\bar{u}^{*2}, \end{aligned}$$

8 $$\begin{aligned}{} & {} \left( \frac{\partial \bar{v^*}}{\partial \bar{r^*}} \bar{u^*}-\frac{\bar{u^*} \bar{v^*}}{\bar{r^*}}+ \frac{\partial \bar{v^*}}{\partial \bar{z^*}} \bar{w^*} \right) =\frac{\mu _nf}{\rho _{nf}}\left( \frac{\partial ^2 \bar{v^*}}{\partial \bar{r}^{*2}}+\frac{1}{\bar{r^*}}\frac{\partial \bar{v^*}}{\partial \bar{r^*}}- \frac{\bar{v^*}}{\bar{r}^2}+ \frac{\partial ^2 \bar{v^*}}{\partial \bar{z}^{*2}}\right) \nonumber \\{} & {} \quad -\frac{\sigma _{nf}}{\rho _{nf}} B^{*2}_{o} \bar{v^*}+\frac{1}{\rho _{nf}}{g^*}\left[ \Lambda _{1} (\bar{T^*}-T^*_{\infty })+(\Lambda _{2} (\bar{T^*}-T^*_{\infty })^{2}\right] -F\bar{v}^{*2}, \end{aligned}$$

9 $$\begin{aligned}{} & {} \rho _{nf}\left( \frac{\partial \bar{w^*}}{\partial \bar{r}}\bar{u^*}+\frac{\partial \bar{w^*}}{\partial \bar{z}} \bar{w}^* \right) =\mu _{nf}\left( \frac{\partial ^2 \bar{w^*}}{\partial \bar{r^2}}+\frac{\partial ^2 \bar{w^*}}{\partial \bar{z^2}}+\frac{1}{\bar{r}}\frac{\partial \bar{w^*}}{\partial \bar{r}}\right) -\frac{\partial \bar{p}}{\partial \bar{z}}, \end{aligned}$$

10 $$\begin{aligned}{} & {} \frac{\partial \hat{T^*}}{\partial \bar{r}} \bar{u^*}+ \frac{\partial \bar{T^*}}{\partial \bar{z^*}}\bar{w^*}= \left( \alpha _{nf} + \frac{16 \sigma _1 \bar{T}^{*3}_\infty }{3\left( \rho Cp\right) _{nf}K^*} \right) \left( \frac{\partial ^{2} \bar{T^*}}{\partial \bar{r}^{*2}}+\frac{1}{\bar{r^*}}\frac{\partial \bar{T^*}}{\partial \bar{r^*}} + \frac{\partial ^{2} \bar{T^*}}{\partial \bar{z}^{*2}}\right) . \end{aligned}$$

The following boundary conditions apply to the problem^34^.

11 $$\begin{aligned}{} & {} \text {when} ~\bar{z^*}=0, \,\,\,\,\,\,\,\,\,\ \bar{w^*}=0, \,\,\,\,\,\,\,\,\,\ \bar{v^*}=\bar{r^*} \bar{\Omega ^*}, \,\,\,\,\,\,\,\,\,\ \bar{u^*} =L^{*}_1\frac{\partial \bar{u^*}}{\partial \bar{z^*}}, \,\,\,\,\,\,\,\,\,\ \bar{T^*}=T^*_{w}, \,\,\,\,\,\,\,\,\,\ \nonumber \\{} & {} \text {when} ~\bar{z^*}\rightarrow \infty , \,\,\,\,\,\,\,\,\,\ ~\bar{u^*} \rightarrow 0, \,\,\,\,\ \bar{T^*} \rightarrow T^*_{\infty }, \,\,\,\,\,\,\,\,\,\ ~\bar{p^*} \rightarrow p^*_{\infty }. \end{aligned}$$

Here ${g^*}$ is the gravitational acceleration, $\bar{p^*}$ indicates pressure, $\Lambda _1$ is mixed convection due to temperature, and $L^{*}_1$ stands for the wall slip ratio in this instance.

The dynamic viscosity of the base fluid $\mu _{nf}$ are considered as follows^16^:

12 $$\begin{aligned} \mu _nf= \frac{\mu }{\left( 1-\phi \right) ^{2.5}}, \end{aligned}$$

The dynamic viscosity of the base fluid $\rho _{nf}$ are considered as follows^7,35^:

13 $$\begin{aligned} \rho _{nf}= & {} \left( 1-\phi \right) \rho _f + \phi \rho _s, \end{aligned}$$

14 $$\begin{aligned} \left( \rho Cp\right) _{nf}= & {} \left( 1-\phi \right) \left( \rho Cp\right) _f + \left( \phi \rho Cp\right) _s, \end{aligned}$$

The following form consideration for thermal conductivity $k_{nf}$ taken from^14,36^:

15 $$\begin{aligned} \frac{k_{nf}}{k_f} = \frac{k_s +\left( m-1\right) k_f - \left( k_f - k_s\right) \phi \left( m-1\right) }{k_s+\left( m-1\right) + \phi \left( k_f-k_s\right) }. \end{aligned}$$

The thermal diffusivity of the nanodfluid $\alpha _{nf}$ are considered as follows^19^:

16 $$\begin{aligned} \alpha _{nf} = \frac{k_{nf}}{\left( \rho Cp\right) _{nf}}. \end{aligned}$$

The previous research failed to acknowledge the intricate relationship between electrical conductivity and volume fraction. Despite the fact that nanoparticles exhibit a significantly higher electrical conductivity than their base liquids, it is well established that the electrical conductivity of the resulting nanofluid will vary as the volume of nanoparticles increases. Hence, the esteemed work of^23^ has deduced the effective electrical conductivity $\sigma _{nf}$.

17 $$\begin{aligned} \frac{\sigma _{nf}}{\sigma _f} = 1 + \frac{3\left( \frac{\sigma _s}{\sigma _f} - 1 \right) \phi }{\left( \frac{\sigma _s}{\sigma _f} + 2 \right) - \left( \frac{\sigma _s}{\sigma _f} - 1 \right) \phi }. \end{aligned}$$

The characteristics of nanoparticles and the base fluid shown in Table 1.

**Table 1:** Thermal and physical aspects of two nanoparticles and water^37,38^.

Nanoparticles/base fluid	TiO$_{2}$	SWCNT	H$_{2}$O
$\rho (kg\, m^{-3})$	4250	2600	997.1
$C_{p}(J\, kg^{-1}.K^{-1})$	686.2	425	4179
$k(W\,m^{-1}.K^{-1})$	8.95	6600	0.6129

The resultant PDEs can be turned into ODEs by using the approximate solution listed here^11^:

18 $$\begin{aligned} \eta =\bar{z^*}\sqrt{\frac{2\bar{\Omega }^*}{\nu _{f}}}, \,\,\,\,\ \bar{u^*} =\bar{r}^* \bar{\Omega }^* \bar{f}'(\eta ), \,\,\,\,\,\ \bar{v^*} = \bar{r^*}\bar{\Omega }^* \bar{g}(\eta ), \,\,\,\,\,\ \bar{w^*} = - \sqrt{2\nu _{f} \bar{\Omega }^*} \bar{f}(\eta ), \nonumber \\ \,\,\ \bar{p}^*=p^*_{\infty }-\mu _f \bar{\Omega }^* P(\eta ), \,\,\,\,\,\ \bar{T}^*= \left( T^*_{w} - T^*_{\infty } \right) \theta (\eta )+T^*_{\infty }. \end{aligned}$$

Although $\bar{f}$, $\bar{g}$ $\theta$, and $\phi$ are non-dimensional variables, $\eta$ is the non-dimensional length through the axis of rotational. Using the similarity transformation ratio from the previous Eqs. (6 and 10) have similar justifications. Eqs. (7, 8, 10) are reorganized as follows:

19 $$\begin{aligned}{} & {} \frac{1}{\left( 1-\phi \right) ^{2.5}\left( 1-\phi +\phi \frac{\rho _s}{\rho _f}\right) }\bar{f}'''+\bar{f}'' \bar{f}-\frac{1}{2}(\bar{f}')^{2}+\frac{1}{2}\bar{g}^{2}-\frac{\frac{\sigma _{nf}}{\sigma _f}}{\left( 1-\phi +\phi \frac{\rho _s}{\rho _f}\right) } M\bar{f}' \nonumber \\{} & {} \quad + \frac{1}{\left( 1-\phi +\phi \frac{\rho _s}{\rho _f}\right) } \chi _T \left( 1+\beta _T \theta \right) \theta -F_r (\bar{f}')^2=0, \end{aligned}$$

20 $$\begin{aligned}{} & {} \frac{1}{\left( 1-\phi \right) ^{2.5}\left( 1-\phi +\phi \frac{\rho _s}{\rho _f}\right) } \bar{g}''+ \bar{g}' \bar{f} -\bar{g} \bar{f}' - \frac{\frac{\sigma _{nf}}{\sigma _f}}{\left( 1-\phi +\phi \frac{\rho _s}{\rho _f}\right) } M \bar{g} +{\left( 1-\phi +\phi \frac{\rho _s}{\rho _f}\right) }\chi _T\nonumber \\{} & {} \left( 1+\beta _T \theta \right) \theta -F_r (\bar{g}')^2=0, \end{aligned}$$

21 $$\begin{aligned}{} & {} \left( \frac{k_{nf}}{k_f} + \frac{4}{3}Rd\right) \theta '' + Pr \left( 1-\phi \right) ^{2.5}\left( 1-\phi +\phi \frac{\rho _s}{\rho _f}\right) \bar{f} \theta ' =0. \end{aligned}$$

The boundary conditions are altered as:

22 $$\begin{aligned}{} & {} \text {when} ~\eta =0, \,\,\,\,\,\,\ \bar{g}=1, \,\,\,\,\,\,\,\ \bar{f}'=\alpha \bar{f}'', \,\,\,\,\,\,\,\ \theta =1, \,\,\,\,\,\,\,\ \bar{f} =0, \end{aligned}$$

23 $$\begin{aligned}{} & {} \text {when}\,\, ~\eta \rightarrow \infty , \,\,\,\,\,\,\ \theta \rightarrow 0, \,\,\,\,\,\,\,\ \bar{f}^{'}\rightarrow 0,\,\,\,\,\,\,\,\ \bar{P}^{*}\rightarrow 0, \,\,\,\,\,\,\,\ \bar{g}\rightarrow 0. \end{aligned}$$

Here prime derivative indicate by $\bar{f}^{'}$ and $\bar{g}^{'}$ with respective to $\eta$, $\alpha =L^*_1 \sqrt{\frac{2{\bar{\Omega }}^*}{\nu _{f}}}$ is stand for velocity slip variable, $P_r =\frac{\nu _f}{\alpha _f}$ denotes the Prandtl number, $\beta _T=\frac{\Lambda _2}{\Lambda _1}\left( T^*_w-T^*_\infty \right)$ is stand for quadratic convection variable due to temperature, $\chi _T=\frac{\bar{g^*} \Lambda _1 \left( \bar{T}^*-T_{\infty )}\right) }{2r{\bar{\Omega }}^{2}\rho _f}$ is a nonlinear mixed convection parameter due to temperature, $Rd= \frac{4\sigma _1 T^{3}_\infty }{K K_f}$ stands for thermal radiation.

It should be noted that nanofluid pressure can be easily determined by integrating Eq. (10). The frictional force acting on a disk with radius R, in accordance with the definite integral, is the value that is of a practical problem in this case.

24 $$\begin{aligned} \bar{T}= & {} -2 r^{2} \pi \int _{0}^{R} \mu _{nf} \frac{\partial V}{\partial \bar{z}}\mid _{z=0}, \nonumber \\ \bar{T}= & {} -\frac{\pi \bar{\Omega }}{2} \frac{1}{\left( 1-\phi \right) ^{2.5}}\sqrt{2\nu _f \bar{\Omega } R^{4}} \bar{g}^{'}\left( 0\right) . \end{aligned}$$

Additionally, the tangential stress $\bar{\tau ^*_r}$ is

25 $$\begin{aligned} \bar{\tau ^*_r}= & {} \mu _{nf}\left( \frac{\partial \bar{u}^{*}}{\partial \bar{z}^{*}}+\frac{\partial \bar{w}^{*}}{\partial \bar{r}^{*}}\right) _{z=0}, \nonumber \\ \bar{\tau ^*_r}= & {} r\bar{\Omega }^{*} \frac{\mu _{f}}{\left( 1-\phi \right) ^{2.5}} \sqrt{\frac{2 \bar{\Omega }^{*}}{\nu _f}}\bar{f}^{''}\left( 0\right) . \end{aligned}$$

Radial stress $\bar{\tau _\theta }$ is

26 $$\begin{aligned} \bar{\tau _\theta }= & {} \mu _{nf}\left( \frac{\partial \bar{v}^{*}V}{\partial \bar{z}^{*}}+\frac{\partial \bar{w}^{*}}{\partial \bar{r}^{*}}\right) _{z=0}, \nonumber \\ \bar{\tau _\theta }= & {} r\bar{\Omega }^{*} \frac{\mu _{f}}{\left( 1-\phi \right) ^{2.5}}\sqrt{\frac{2 \bar{\Omega }^{*}}{\nu _f}}\bar{g}^{'}\left( 0\right) . \end{aligned}$$

which results in the subsequent skin friction coefficient:

27 $$\begin{aligned} \bar{C_f}= & {} \frac{\sqrt{\bar{\tau }^{*2}_r + \bar{\tau }^{*2}_\theta }}{\rho _{nf} r^{2} \bar{\Omega }^{2}}, \nonumber \\ \bar{C_f}= & {} \frac{1}{\left( 1-\phi \right) ^{2.5}} \,\,\,\ Re^{\frac{-1}{2}} \,\,\,\ \sqrt{\bar{f}^{''}\left( 0\right) ^{2}+|\bar{g}^{'}\left( 0\right) ^{2}}. \end{aligned}$$

In order to calculate the local Nusselt number $\bar{Nu}_r$, we add the heat fluxes caused by nanoparticle diffusion and conduction,

28 $$\begin{aligned} \bar{Nu}_r=\frac{rq^{''}}{K\left( T^{*}_w - T^{*}_\infty \right) }. \end{aligned}$$

The zero wall mass flux supposition, it should be emphasized, removes the wall heat flux caused by nanoparticle diffusion. Eq. 28 does have the form given by Eq. 18 where the disk’s heat flow is present is $q^{''}=-k\frac{\partial \bar{T^{*}}}{\partial \bar{z}^{*}}$.

29 $$\begin{aligned} \bar{Nu}_r=-\frac{k_{nf}}{k_f} \,\,\ Re^{\frac{1}{2}} \,\,\,\,\ \theta ^{'}\left( 0\right) . \end{aligned}$$

## Numerical solution

Here, a novel shooting technique and a direct discretization strategy have been innovated in order to tackle boundary value quandaries. It is imperative to employ an infallible, high-order approach that can proficiently surmount general, nonlinear boundary value issues. Fortunately, the RK method, a user-friendly and advantageous resource offered by MATLAB, can competently address relatively intricate conundrums. The method employs an iterative framework that effectively solves nonlinear systems of equations. Let’s replace $Z_1=\bar{f}$, $Z_2=\bar{f}^{'}$, $Z_3=\bar{f}^{''}$, $Z_4=\bar{g}$, $Z_5=\bar{g}^{'}$, $Z_6=\theta$, $Z_7=\theta ^{'}$. Thus, the ordinary differential equations Eqs. (19–21) along with boundary conditions Eqs. (22, 23) can be written as:

30 $$\begin{aligned} Z^{'}_{1}= & {} Z_{2}, \end{aligned}$$

31 $$\begin{aligned} Z^{'}_{2}= & {} Z_{3}, \end{aligned}$$

32 $$\begin{aligned} Z^{'}_{3}= & {} \left( 1-\phi \right) ^{2.5}\left( 1-\phi +\phi \frac{\rho _s}{\rho _f}\right) \nonumber \\{} & {} \left( \frac{1}{2} Z^{2}_{2} - \frac{1}{2} Z^{2}_{4} - Z_{1} Z_{3} +\frac{\frac{\sigma _{nf}}{\sigma _f}}{\left( 1-\phi +\phi \frac{\rho _s}{\rho _f}\right) } M Z_2 - \frac{1}{\left( 1-\phi +\phi \frac{\rho _s}{\rho _f}\right) } \chi _T \left( 1+\beta _T Z_6 \right) Z_6 -F_r Z^2\right) , \end{aligned}$$

33 $$\begin{aligned} Z'_4= & {} Z_3, \end{aligned}$$

34 $$\begin{aligned} Z^{'}_{5}= & {} \left( 1-\phi \right) ^{2.5}\left( 1-\phi +\phi \frac{\rho _s}{\rho _f}\right) \nonumber \\{} & {} \left( Z_4 - Z_1 s_5 + \frac{\frac{\sigma _{nf}}{\sigma _f}}{\left( 1-\phi +\phi \frac{\rho _s}{\rho _f}\right) } M Z_4 - {\left( 1-\phi +\phi \frac{\rho _s}{\rho _f}\right) } \chi _T\left( 1+\beta _T Z_6 \right) Z_6-F_r Z^2\right) , \end{aligned}$$

35 $$\begin{aligned} Z^{'}_{6}= & {} Z_{7}, \end{aligned}$$

36 $$\begin{aligned} Z^{'}_{7}= & {} \frac{1}{\left( \frac{k_{nf}}{k_f} + \frac{4}{3}Rd\right) }\left( -Pr\left( 1-\phi \right) ^{2.5} \left( 1-\phi +\phi \frac{\rho _s}{\rho _f}\right) Z_1\right) . \end{aligned}$$

along with the boundary conditions:

37 $$\begin{aligned}{} & {} \text {at}\,\ \eta =0, \,\,\,\,\,\ Z_{4}=1, \,\,\,\,\,\ Z_{2}= \alpha _1 Z_3, \,\,\,\,\,\ Z_{6}= 1, \,\,\,\,\,\,\ Z_{z}=0, \,\,\,\,\,\,\ \end{aligned}$$

38 $$\begin{aligned}{} & {} \text {at}\,\ ~\eta \rightarrow \infty \,\,\,\ Z_6'\rightarrow 0,\,\,\,\ Z_2\rightarrow 0, \,\,\,\ P\rightarrow 0, \,\,\,\ Z_4\rightarrow 0. \end{aligned}$$

### Verification

We verified the results of skin friction and Nusselt number with Yin et al.^34^ and Acharya^37^ in order to confirm the accuracy of our most recent research, with $Pr=6.2$ (water at 300 K) and set all other variables to zero. There is a high degree of agreement with the body of literature, as seen in Table 2.

**Table 2:** A comparison of $f'(0)$ and $-\theta ^{'}(0)$ for $Pr=6.2$.

	Yin et al.^34^	Acharya et al.^37^	Our outcomes
$\bar{f}'(0)$	0.51022941	0.5102295	0.510229497
$-\theta ^{'}(0)$	0.93387285	0.9338728	0.933872789

## Result and discuusion

**Figure 2:**
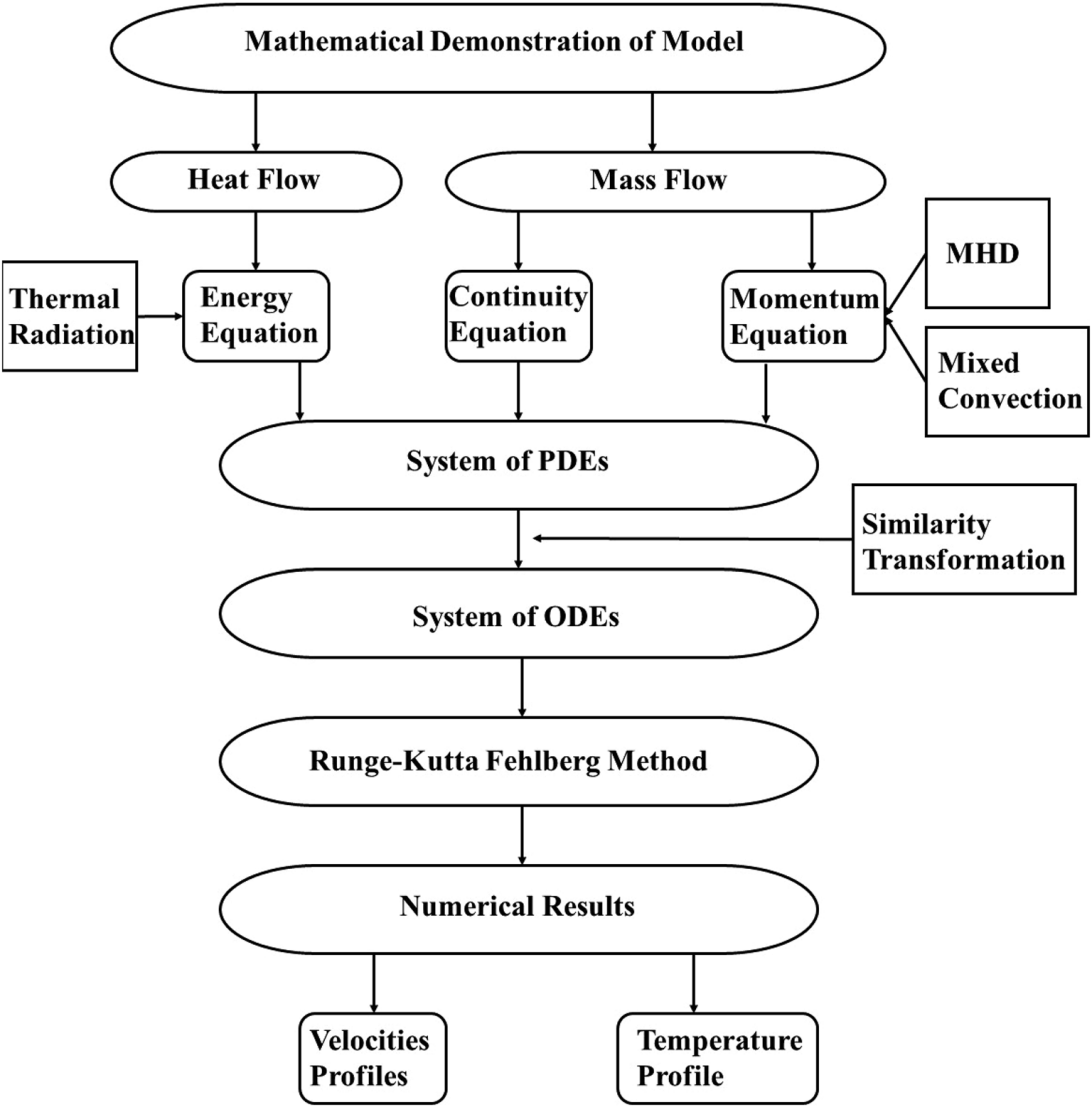
Schematic representation of the current study.

**Figure 3:**
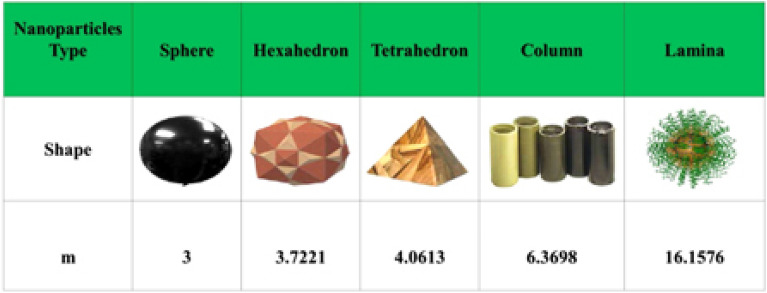
Shape factors for various particles^39^.

Some significant results are obtained by solving the transform Eqs. (19-21) along boundary conditions (22, 23) are described in this section. Runge-Kutta of order four is the technique used for numerical solutions. Extensive computational work is done to determine the effects on $\bar{f}^{'}$, $\bar{g}$, and $\theta$ along with the variational behavior of parameters. The flow chart of the current study is shown in Fig. 2. Figure 3 shows that the shape factor *m* depends on the various particle shapes^39^. In this context, the term “shape factor” refers to a value that is influenced by an object’s shape but unaffected by its dimensions.

Table 3 shows the numerical calculation of skin friction for TiO$_{2}$/H$_{2}$O and SWCNT/H$_{2}$O nanofluid with various parameters. It can be noticed that the value of skin friction for TiO$_{2}$/H$_{2}$O and SWCNT/H$_{2}$O increases with the high value of the magnetic parameter and Darcy Forchheimer are increased but decreased with mixed convection variable. Table 4 shows the computational calculation of the Nusselt number for TiO$_{2}$/H$_{2}$O and SWCNT/H$_{2}$O nanofluid with various parameters. It can be noticed that the value of Nusselt number for TiO$_{2}$/H$_{2}$O and SWCNT/H$_{2}$O decrease with the high value of the magnetic parameter, Darcy Forchheimer, and thermal radiation parameter while it has increasing behavior for different nanoparticles shape factors. Table 4 shows that the enhancement in heat transfer rate is greatest for laminar shape nanoparticles and least for sphere shape nanoparticles.

**Figure 4:**
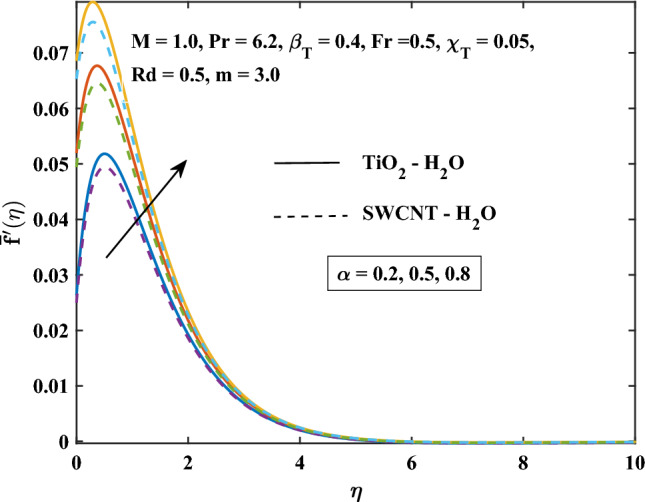
Velocity slip parameter influence on the primary velocity.

**Figure 5:**
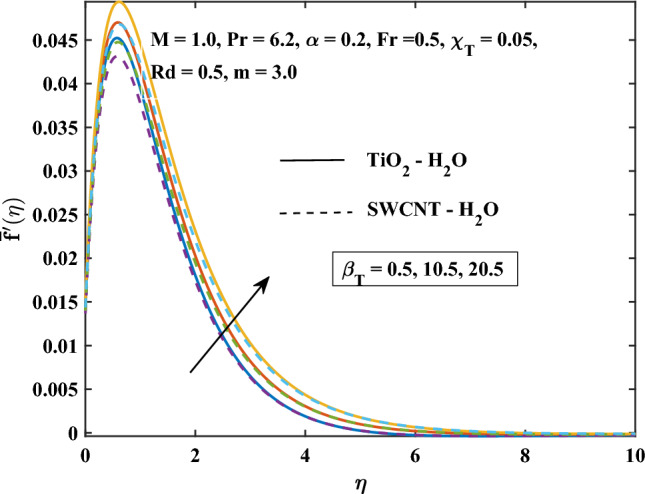
Mixed convection parameter influence on the primary velocity.

**Figure 6:**
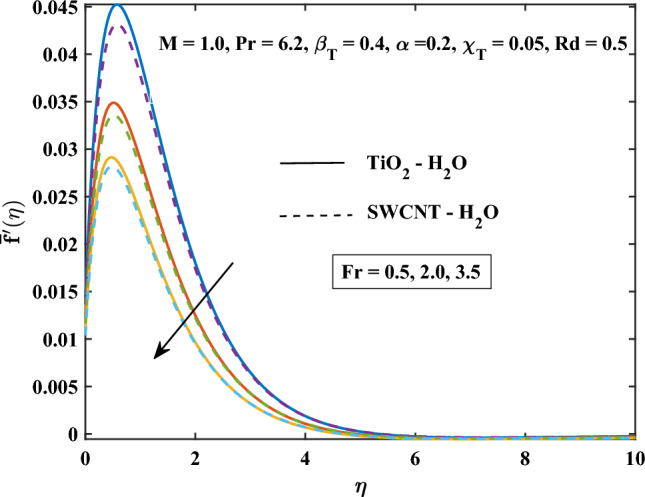
Darcy Forchiemer parameter influence on the primary velocity.

**Figure 7:**
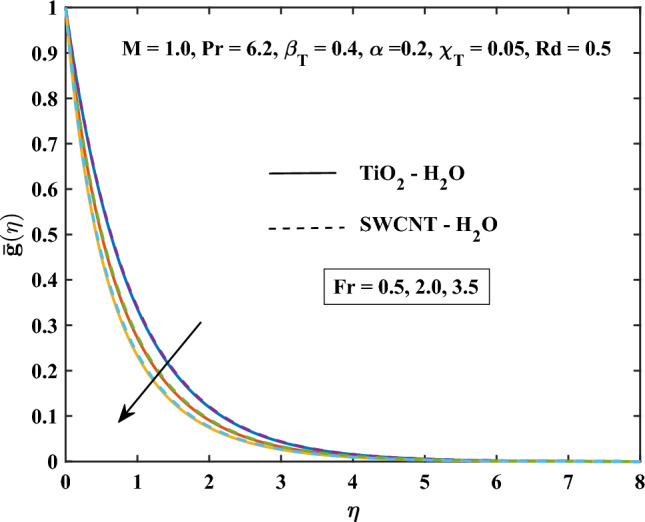
Darcy Forchiemer parameter influence on the secondary velocity.

**Figure 8:**
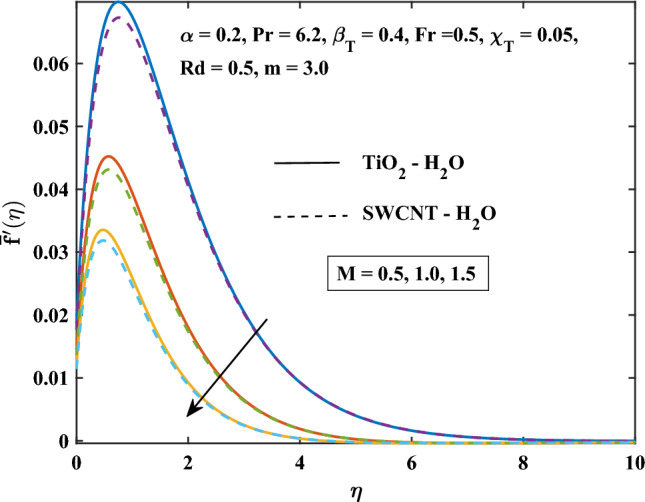
Magnetic parameter influence on the primary velocity.

**Figure 9:**
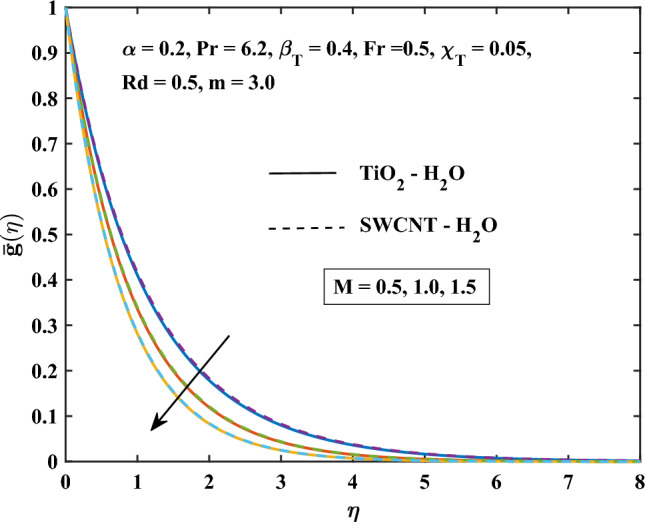
Magnetic parameter influence on the secondary velocity.

The velocity profiles against different values of parameters are shown in Figs. 4, 5, 6, 7, 8 and 9. Figure 4 shows the velocity profile for various inputs of $\alpha _1$ with TiO$_{2}$/H$_{2}$O and SWCNT/H$_{2}$O nanofluids. Increase in $\alpha _1$ raises $\bar{f'}$. At a specific increment in $\alpha _1$, $\bar{f'}$ rises gradually with an increase in $\eta$ and reaches its maximum value at a predetermined distance. This is because the interaction between the liquid and solid phases within the nanofluid is directly influenced by the velocity slip variable. Figure 5 shows the primary velocity of TiO$_{2}$/H$_{2}$O and SWCNT/H$_{2}$O nanofluid are increase with the increasing of $\beta _T$.The increase in mixed convection variable $\beta _T$ for different values, the velocity profile of TiO$_{2}$/H$_{2}$O and SWCNT/H$_{2}$ Onanofluid enhance. This is due to the fact that when the buoyancy on free convection gets significant, mixed convection occurs. A higher mixed convection parameter $\beta _T$ indicates that natural convection (buoyancy) will have a greater impact. The difference in density brought on by temperature gradients tends to promote fluid motion during natural convection. Stronger temperature gradients may result from this, and then the primary velocity increases. Figures 6, 7 explain how the velocities profile varies for different values of *Fr* in comparison to the TiO$_{2}$/H$_{2}$O and SWCNT/H$_{2}$O nanofluid. Both components of velocity decrease with the increase of *Fr* because of the inertial resistance, which is shown by a higher Darcy-Forchheimer value. Figures 8, 9 show the graph of velocities with magnetic variables. It can be noticed that both velocities fall in the TiO$_{2}$/H$_{2}$O and SWCNT/H$_{2}$O nanofluid with the enhancement of magnetic variable *M*. This is due to the fact that the Lorentz force is imposed by the presence of a transverse magnetic field, which impedes the velocity field. The velocity profiles decrease as a result of the retarding force rising in tandem with the values of *M*.

**Table 3:** Skin friction coefficient numerical results against various parameter inputs.

*M*	*Fr*	$\beta _{T}$	$Re^{0.5}\bar{C_{f}}$	$Re^{0.5}\bar{C_{f}}$
			TiO$_{2}$-H$_{2}$O	SWCNT-H$_{2}$O
0.6			1.1008	1.0840
1.0			1.2721	1.2588
1.4			1.4286	1.4173
0.5	0.4		1.2399	1.2280
	0.9		1.3943	1.3754
	1.4		1.5341	1.5092
	0.5	0.3	1.2722	1.2587
		0.7	1.2719	1.2583
		1.1	1.2716	1.2580

**Table 4:** Nusselt number numerical results against various parameter inputs.

*M*	*Fr*	*Rd*	*m*	$Re^{-0.5}\bar{Nu}_r$	$Re^{-0.5}Nu$
				TiO$_{2}$-H$_{2}$O	SWCNT-H$_{2}$O
0.6				0.2990	0.2920
1.0				0.2159	0.2084
1.4				0.1649	0.1589
0.5	0.4			0.2201	0.2123
	0.9			0.2012	0.1947
	1.4			0.1863	0.1808
	0.5	0.5		0.2397	0.2310
		1.0		0.1890	0.1828
		1.5		0.1586	0.1540
		0.7	3.0	0.2084	0.2159
			3.7221	0.2117	0.2181
			4.0613	0.2132	0.2191
			6.3698	0.2232	0.2247
			16.1576	0.2604	0.2378

**Figure 10:**
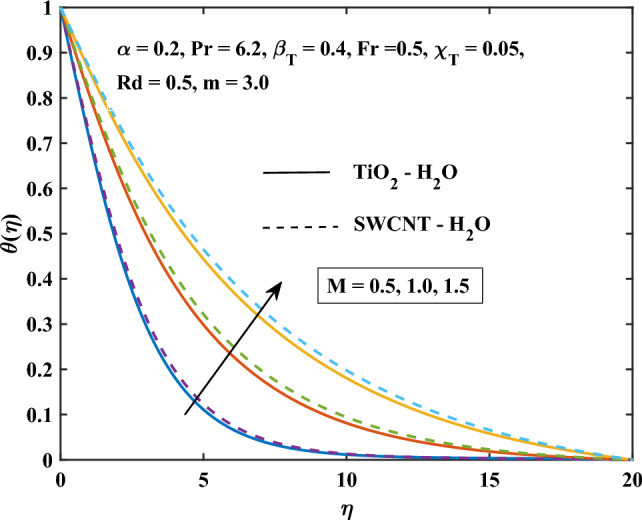
Magnetic parameter influence on the temperature.

**Figure 11:**
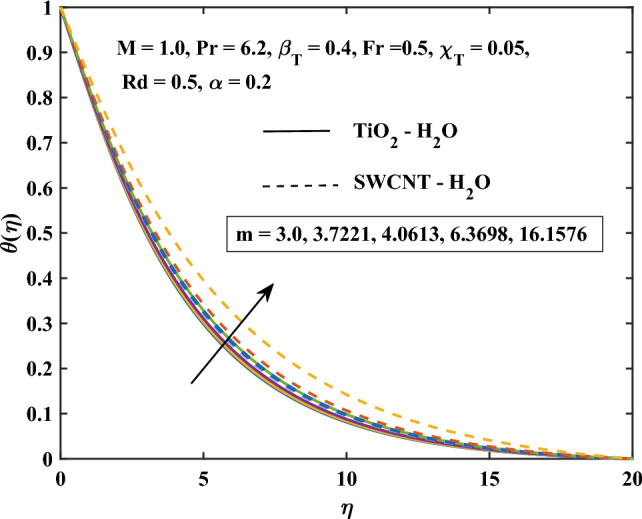
Shape factor influence on the temperature.

**Figure 12:**
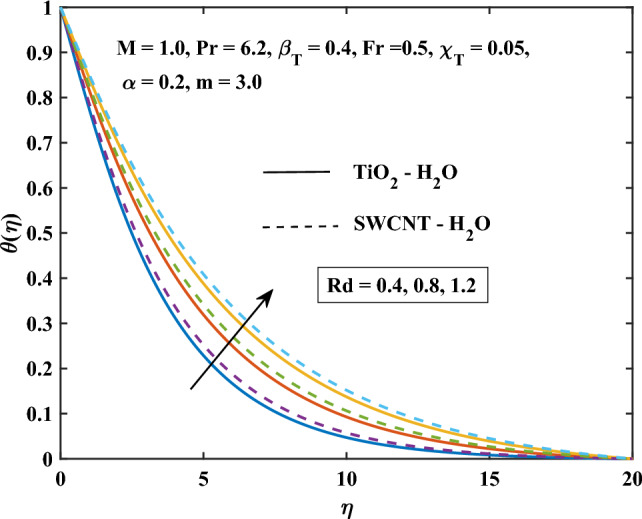
Thermal radiation parameter influence on the temperature.

**Figure 13:**
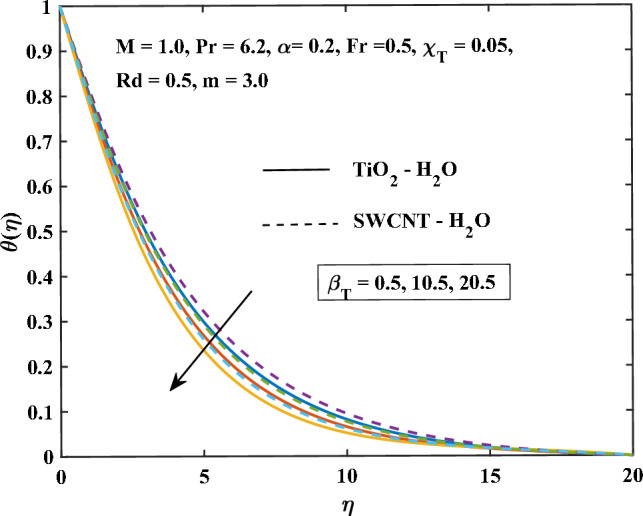
Mixed convection parameter influence on the temperature.

**Figure 14:**
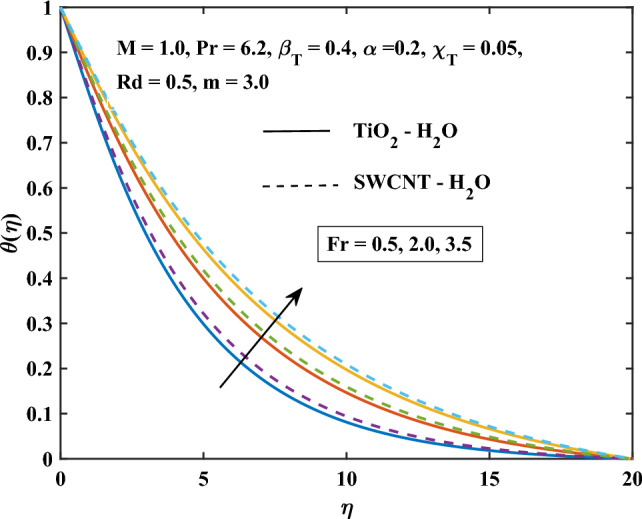
Darcy Forchiemer parameter influence on the temperature.

The velocity profiles against different values of parameters are shown in Figs. 10, 11, 12, 13 and 14. Figure 10 shows the magnetic parameter effect on the temperature profile. It can be noticed that with the high values of the magnetic variable *M*, the temperature-enhanced in SWCNT/H$_{2}$O and TiO$_{2}$/H$_{2}$O nanofluids. This is due to the opposing Lorentz force, which crosses the fluid motion and generates heat. Figure 11 shows the temperature profile graph with the shape factor *m*. It can be noticed that the profile of SWCNT/H$_{2}$O and TiO$_{2}$/H$_{2}$O nanofluids are increase with the increasing of *m*. Figure 11 shows the influence of various nanoparticle shapes on the nanofluid’s capacity for heat transmission within the boundary layer of nanofluid flow. The relationship between the shape factor’s various values and the temperature distribution is demonstrated for SWCNT/H$_{2}$O and TiO$_{2}$/H$_{2}$O indicates how the nanofluid’s non-dimensional temperature increases as *m* increases. Due to their increased surface area’s tendency to absorb more heat from the boundary layer than the other shapes, spherical shape nanoparticles are found to have the lowest temperature. This explains why spherical nanoparticles transfer heat at the border faster than the other shapes. For the spherical-shaped nanoparticles, this explains why the boundary has the maximum rate of heat transmission. For nanoparticles, the laminar shape shows the greatest temperature. Figure 12 shows the graph of thermal radiation with a temperature of TiO$_{2}$/H$_{2}$O and SWCNT/H$_{2}$O nanofluids. The temperature profile of SWCNT/H$_{2}$O and TiO$_{2}$/H$_{2}$O nanofluids enhanced with the high values of *Rd*. This is due to the system’s energy being increased by radiation, which leads to intermolecular interactions and changes the system’s temperature and nanoparticle intensity.

Figure 13 shows the temperature profile of TiO$_{2}$/H$_{2}$O and SWCNT/H$_{2}$O nanofluids are increased with the increasing of $\beta _T$. The increase in mixed convection variable $\beta _T$, the temperature is enhanced in SWCNT/H$_{2}$O and TiO$_{2}$/H$_{2}$O nanofluids. This is because natural convection is more likely to occur when there is a variation in density due to temperature differences. This may lead to stronger temperature gradients. Figure 14 shows the effect on the temperature of the increasing values of *Fr*. A higher Darcy-Forchheimer parameter, increases the flow resistance which can hinder the flow of heat and result in reduced heat transfer rates. It can be noticed that more temperature enhanced in SWCNT/H$_{2}$O and TiO$_{2}$/H$_{2}$O nanofluids increase with the increasing values *Fr*. It is analyzed that from all the temperature profiles with different parameters, the temperature profile of SWCNT/H$_{2}$O is more enhanced in the comparison of TiO$_{2}$/H$_{2}$O.

## Conclusion

In this study, behold the presentation of the intricate mathematical computations that govern the flow of nanofluid produced by a rough rotating disk. The PDE system makes up the physical issue in this paper. In view of the significant nonlinearity, a suitable similarity transformation is used, and an ODEs system with BCs is created. The RK method with MATLAB is used along with a numerical shooting technique to handle these ODEs.

The results to be noted are:

- As the magnetic variable increases the primary, secondary velocity, and Nusselt number components decrease.
- The primary flow rate increases with the increase of the velocity slip variable.
- The temperature profile and skin friction coefficient are enhanced by increasing the magnetic parameter and shape factor values.
- The magnitude of the primary velocity and secondary velocity reduces, and the temperature profile is enhanced with the progressing inputs of *Fr*.
- The increasing inputs of *Rd*, the temperature profiles of SWCNT/H$_{2}$O more prominent in the comparison of TiO$_{2}$/H$_{2}$O.
- The velocity increase, the temperature, and the skin friction coefficient are reduced by increasing inputs of mixed convection variables $\beta _T$ due to temperature.
- The enhancement in heat transfer rate is maximum in laminar nanoparticles shape and the minimum heat enhancement is observed in a spherical shape.

### Future direction

This work can be extended for different non-Newtonian nano, hybrid, and ternary nanofluids flow across the rotating disk. Moreover, this can be extended for the modified Buongiorno model.

## Data Availability

The data that support the findings of this study are available from the corresponding author upon reasonable request.

## List of symbols

$\rho _{f}$: Base fluid density
$\nu _{nf}$: Dynamic viscosity of nanofluid
*M*: Magnetic parameter
$$(\bar{u}^*, \bar{v}^*, \bar{w}^*)$$: Velocity components
*g*: Dimensionless secondary velocity
*Pr*: Prandtl number
$$\bar{Nu}^*_{r}$$: Nusselt number
$\alpha _{nf}$: Thermal diffusivity of nanofluid
$$\theta$$: Dimensionless temperature
$\Lambda _1$ , $\Lambda _2$: Mixed convection due to temperature
$\alpha _{nf}$: Thermal diffusivity of nanofluid
$\nu _{nf}$: Kinematic viscosity of nanofluid
${T^*}_{\infty }$: Temperature away from the surface
*Rd*: Radiation parameter
$\bar{T}^*$: Non-dimensional temperature
$\bar{f}'$: Dimensionless primary velocity
$\bar{L^*_1}$: Wall slip coefficient
*m*: Nanoparticles shape factors
$\alpha$: Velocity slip coefficient
*V*: Velocity in vector form
$\bar{g}'$: Dimensionless secondary velocity
$$\eta$$: Dimensionless variable
$$\rho C_{p}$$: Heat capacity
$K_{nf}$: Thermal conductivity of nanofluid
$F_{r}$: Darcy forchheimer coefficient
$\beta _{T}$: Quadratic convection parameter
$$\bar{t}^*_{r}$$: Tangential stress
$$\bar{t}^*_{\theta }$$: Radial stress
$(\bar{r^*}, \varphi , \bar{z^*})$: Spherical coordinates $\bar{\Omega ^*}$ angular velocity
